# An experimental design for obtaining DNA of a target species and its diet from a single non‐invasive genetic protocol

**DOI:** 10.1002/ece3.10616

**Published:** 2023-10-22

**Authors:** Shrutarshi Paul, Naama Shahar, Merav Seifan, Shirli Bar‐David

**Affiliations:** ^1^ Mitrani Department of Desert Ecology, Blaustein Institutes for Desert Research Ben‐Gurion University of the Negev Midreshet Ben‐Gurion Israel

**Keywords:** diet of herbivores, DNA extraction, *Equus hemionus*, metabarcoding, non‐invasive genetics, trnL

## Abstract

Next‐generation sequencing technology has enabled accurate insights into the diet of wildlife species. The protocols for faecal sample collection and DNA extraction for diet analysis have differed from those focusing on target species, even in most studies combining questions on both aspects. We designed an experiment to evaluate two protocols using 11 parameters and select a single one that will generate both target species (Asiatic wild ass, *Equus hemionus*, in Israel) and diet DNA, as an effective strategy to minimise time, effort, and cost without hampering efficiency. In Protocol A, we swabbed the outer surface of faecal boluses and extracted DNA using a Stool Kit, while for Protocol B, we homogenised faecal matter from inside the bolus followed by extraction using a Powersoil Kit. Protocol A performed significantly better for four parameters, which included, for the target species, microsatellite amplification success and the quantity of the GAPDH gene; and for its diet, the number of exact sequence variants (ESVs) obtained at genus level and plant genus richness. However, there was no significant difference in the amplification success of sex‐linked and plant markers, total reads at genus level, number of genera obtained and plant genus composition. Although we chose Protocol A, both protocols yielded results for the target species and its diet, demonstrating that one single protocol can be used for both purposes, although a pilot study is recommended to optimise the protocol for specific systems. This strategy may also be useful for studies combining target species and their gut microbiome and parasitic load.

## Graphical abstract

In this study, we proposed and demonstrated a framework for selecting one common protocol for evaluating DNA of a species and its diet. We compared between commonly used non‐invasive protocols by evaluating different measures of DNA quality and quantity for the focal species (e.g. amplification success of sex‐linked and microsatellite markers) and for its diet (e.g. number of reads of a chloroplast barcoding marker, trnL and plant genus richness and diversity). We demonstrated that one single protocol can be used for both purposes in ‘combined studies’, but a pilot study is recommended to optimise the protocol for specific systems, as an effective strategy to minimise time, efforts and cost without hampering efficiency.

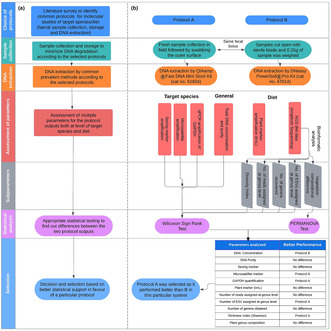

## 1 INTRODUCTION

Molecular scatology has enabled wildlife researchers to answer several questions about population structure, species distribution, dispersal patterns, demographic parameters, genetic diversity and disease assessment using a non‐invasive genetic approach (e.g. Banks & Piggott, 2022; Beja‐Pereira et al., 2009; Goossens & Bruford, 2009; Rodriguez‐Castro et al., 2022; Saranholi et al., 2017; Schilling et al., 2022; Taberlet & Waits, 1999; Waits & Paetkau, 2005; Zemanova, 2021). The scope of molecular scatology has been significantly broadened by the recent advancements in high‐throughput next‐generation sequencing (NGS) approaches (e.g. Bohmann et al., 2014; Dunisławska et al., 2017; Kartzinel et al., 2015; Khan & Tyagi, 2021; Liu et al., 2021; Scasta et al., 2019; Zahedi et al., 2022). One such application of NGS that has gained notable attention recently is the assessment of an animal species' (defined as target species henceforth) fine‐scale dietary spectrum (e.g. Crisol‐Martínez et al., 2016; Czernik et al., 2013; Galan et al., 2018; Liu et al., 2021). This has led to molecular scatology studies that have focused on the diet of target wildlife species (e.g. Anderson et al., 2018; Berman & Inbar, 2022; Henger et al., 2022; Jorns et al., 2020; Khalatbari et al., 2022; King & Schoenecker, 2019; Klure et al., 2022; McLennan et al., 2022; Mitchell et al., 2022; Nelms et al., 2019; O'Rourke et al., 2021; Roffler et al., 2021; Wanniarachchi et al., 2022). In parallel, other molecular scatology studies continue to focus on the behaviour, gene flow and space‐use patterns of the target species itself (e.g. Ambarlı et al., 2018; Baena et al., 2020; Bischof et al., 2016; Biswas et al., 2022; De et al., 2021; Kaczensky et al., 2011; Kefena et al., 2021; King et al., 2021; Paul, Saha, et al., 2023; Paul, Shahar, et al., 2023a, 2023b; Putnová et al., 2019; Renan et al., 2018).

The protocols for collecting, preserving and extracting DNA, adopted by studies that focus on dietary assessments, have mostly been different from those that concentrate on the target species itself (e.g. Craine, 2021; Kawamoto et al., 2013; Kovach et al., 2003; Modi et al., 2021; Pansu et al., 2019). This trend has also continued in the majority of recent molecular scatology studies that have started to combine questions on the target species itself (gene flow, inbreeding, sex ratio and dispersal patterns) with questions regarding their diet (e.g. Abdullah‐Fauzi et al., 2022; Bison et al., 2015; Buglione et al., 2018; Jesmer et al., 2020; Shi et al., 2021; Shin et al., 2020) although a recent study has demonstrated utilities of one single protocol (de Flamming et al., 2023). The collection, processing and extraction of faecal samples for target species studies often revolve around collecting samples and storing them in either −20°C conditions or in a storage medium such as ethanol, silica beads or RNAlater solution, followed by swabbing/scraping off the outer layer and extracting DNA using stool kit‐based methods/CTAB method/phenol‐chloroform (Ball et al., 2007; Biswas et al., 2019; Paul et al., 2019; Ramón‐Laca et al., 2015; Waits & Paetkau, 2005). In the case of studies that focus on diet assessment, part of the sample is weighed and homogenised, and then, the DNA is extracted by kit‐based extraction methods (stool kit/soil kit) (Ando et al., 2020; Craine, 2021; Craine et al., 2016; Guo et al., 2018; Vo & Jedlicka, 2014). The different protocols specified for target species or diet may be advantageous when conducting these studies independently; however, when aiming at collecting both target species population data and dietary information simultaneously in one study, separate protocols are not time or cost‐effective. Instead, an efficient common protocol, used for both purposes, could advance such integrated projects, by saving time, money and effort. The potential of such combined protocols has been investigated in a recent study (de Flamingh et al., 2023).

The success rate of non‐invasive genetic methods is known to be affected by various factors such as field conditions, the presence of PCR inhibitors coming from diet, time of exposure to environment before collection, and faecal sample storage and processing, among other factors (Agetsuma‐Yanagihara et al., 2017; Bi et al., 2014; Broquet et al., 2007; Frantzen et al., 1998; Kovach et al., 2003; Murphy et al., 2000, 2002; Nsubuga et al., 2004; Wultsch et al., 2015). As a result, the efficiency of a particular non‐invasive protocol may vary with species, habitats and climatic conditions, and even within different populations of the same species. Thus, each given system may have its most suitable protocol (Ando et al., 2020; Beja‐Pereira et al., 2009; Renan et al., 2012). Therefore, before starting a full‐scale non‐invasive ‘combined study’, an optimisation to search for the most efficient method during a pilot study, with system‐specific samples, should be performed.

**FIGURE 1:**
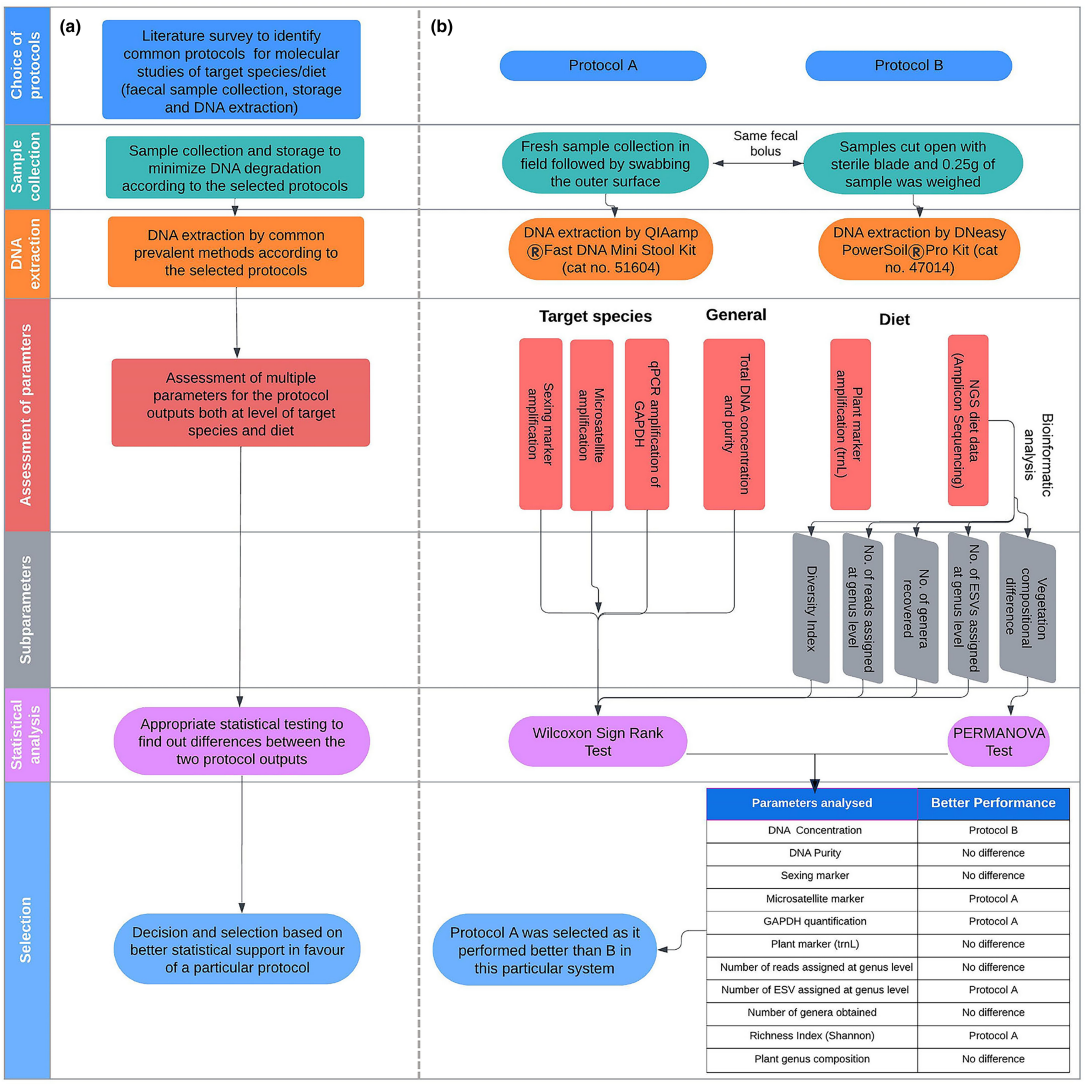
Schematic workflow for selection of a combined protocol for obtaining DNA of a target species and its diet. Panel a: General step‐by‐step recommendations for a pilot study designed to select a combined protocol. Panel b: Demonstration of the recommendation through our case study on wild ass and its diet.

Here, we describe a methodological framework (Figure 1) for conducting a pilot study aimed at identifying a common protocol for collecting and extracting DNA from faecal samples that will generate data both on target species and its diet in a specific system. We demonstrate the framework by examining the Asiatic wild ass (*Equus hemionus*), as the target species, and its diet within the Negev Highlands in Israel and comparing between two commonly used non‐invasive genetic protocols. One of the protocols has mainly been used for studies on target species (e.g. Alberts et al., 2017; Angom et al., 2020; Bach et al., 2022; Biswas et al., 2019; Bourgeois et al., 2019; Modi et al., 2021; Natesh et al., 2019; Ramón‐Laca et al., 2015), while the other has mostly been used in dietary studies (e.g. Alberdi et al., 2018; Ando et al., 2020; Craine, 2021; Craine et al., 2015, 2016; Jesmer et al., 2020; Jorns et al., 2020; Pansu et al., 2019; van Zinnicq Bergmann et al., 2021). In the first protocol (Protocol A), we collected fresh faecal samples and then swabbed the outer surface in the field and extracted DNA in the laboratory using the QIAamp Fast DNA Mini Stool Kit method. In the second protocol (Protocol B), we homogenised faecal matter obtained from inside the faecal boluses of the same samples and extracted DNA using the DNeasy Power Soil Pro Kit method. We then compared the two protocols by checking and evaluating the quality and quantity of the extracted DNA for both the target species and its diet using the following parameters: For assessment of the target species' DNA extracted by Protocols A and B, we evaluated the amplification success of sex‐chromosome markers and microsatellite markers through a gel‐based assay and quantified the amplification of a housekeeping mammalian gene, GAPDH, through the qPCR technique. For assessment of the DNA of the diet extracted by both protocols, we calculated the amplification success of a chloroplast barcoding marker, trnL (Taberlet et al., 2006), through a gel‐based assay, and we quantified the number of total reads assigned to a genus, the number of exact sequence variants (ESVs) assigned to a genus, the number of identified plant genera, diversity indices at the genus level and the relative read abundances of each genus through a metabarcoding technique. We believe such a methodological framework may benefit similar studies dealing with other wildlife species, specifically mammalian herbivores, to optimise the best protocol for ‘combined studies’.

## 2 MATERIALS AND METHODS

### 2.1 Study species

**FIGURE 2:**
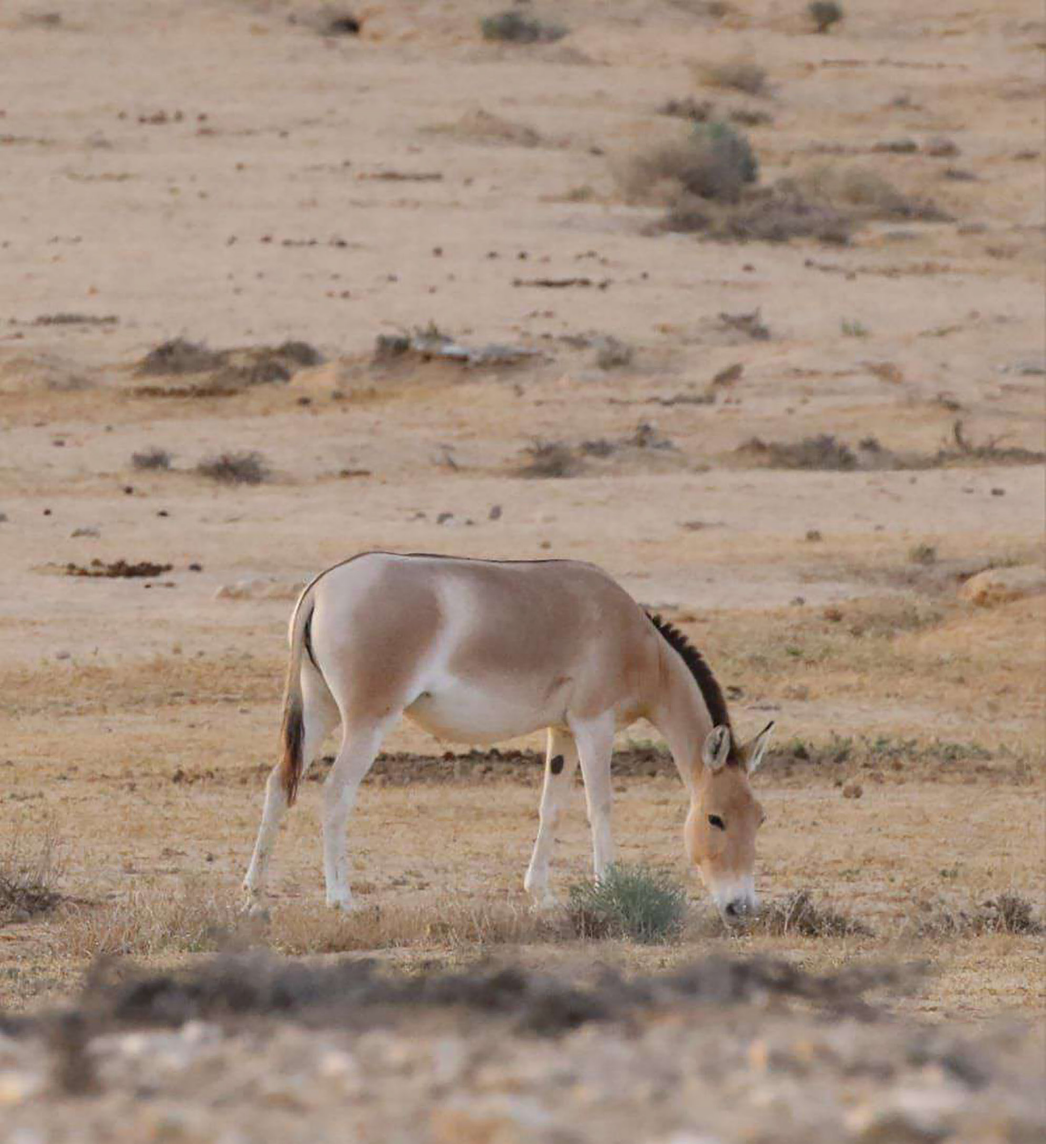
Asiatic wild ass (*Equus hemionus*) grazing in the Negev Highlands of Israel. (Picture taken by Dr. Krista Oswald).

This study focused on the reintroduced population of the Asiatic wild ass (*Equus hemionus* spp.) (Figure 2), a large herbivore in Israel's Negev desert (Saltz et al., 2000; Saltz & Rubenstein, 1995). The current Asiatic wild ass population in Israel originated from a captive breeding core established at the Hai‐Bar Yotvata Reserve in 1968, by six individuals belonging to the *E. h. onager* sub‐species and five individuals of *E. h. kulan* (Saltz et al., 2000). Subsequently, the population has increased to an estimated >250 individuals (Gueta et al., 2014; Zecherle et al., 2020). They are distributed throughout the Negev desert, but their space‐use patterns are affected by water sources, topography, microhabitat climatic conditions and forage availability (Giotto et al., 2015; Nezer et al., 2017). Globally, they are categorised as ‘Near Threatened’ by IUCN (Kaczensky et al., 2020) but locally are listed as ‘Endangered’ and protected by law in Israel (Meiri et al., 2019).

### 2.2 Faecal sample collection, storage, and processing according to selected protocols

We conducted our faecal sampling opportunistically in the Negev highlands during spring, when flowering season is at its peak in Israel (Figure S1). We collected fresh samples (wet outside and inside) as their DNA quality is better than old samples (Hájková et al., 2004; Nsubuga et al., 2004) (Figure S1). As Asiatic wild ass faeces generally occur as an aggregate of round boluses, we collected one bolus from the middle layer to avoid contamination from the air and soil. Each bolus was processed by two different protocols, with each method being independent of the other. For Protocol A, we carefully swabbed the outer layer of the single bolus/pellet using a sterile cotton swab wet with Inhibitex buffer and stored the swab in an Eppendorf tube containing 600 μL of Inhibitex buffer at 4°C in the field and later at −20°C in the laboratory (Ramón‐Laca et al., 2015; Renan et al., 2018). After swabbing, the faecal bolus was kept in a sterile Ziploc bag and stored at 4°C. In the laboratory, for Protocol B, the same faecal bolus was cut with a sterile blade, and 0.25 g of faecal matter from the inside was weighed and stored at −20°C until further processing.

### 2.3 DNA extraction according to selected protocols and quantification of DNA

#### 2.3.1 Protocol A‐QIAamp® Fast DNA Mini Stool Kit (cat no. 51604)

We used the kit with a slightly modified protocol in which collected faecal swabs were flipped inside Eppendorf tubes using tweezers and centrifuged for 3 min to squeeze buffer into the tube. Swabs were then discarded, and subsequent extraction was completed following the standard spin‐column protocol from the DNA stool kit (QIAGEN). Finally, the DNA was eluted in 100 μL of ATE buffer and stored at −20°C.

#### 2.3.2 Protocol B‐DNeasy PowerSoil® Pro Kit (cat no. 47014)

The faecal contents (0.25 g) were homogenised and lysed, followed by extraction using the DNeasy PowerSoil® Pro Kit (QIAGEN) according to the manufacturer's protocol (Craine, 2021). Finally, the DNA was eluted in 100 μL of elution buffer and frozen at −20°C.

The quality (absorbance 260/280) and quantity (DNA concentration) of the DNA from the two protocols were assessed using a NanoDrop™ One spectrophotometer (Thermo Fisher Scientific).

### 2.4 Assessment of multiple parameters of the two protocol outputs at the level of target species DNA

#### 2.4.1 Amplification success of sex‐linked and microsatellite markers through PCR amplification

We assessed the quality and quantity of Asiatic wild‐ass DNA by amplifying specific genetic markers. We amplified a ZFX‐ZFY (553–604 bp) sexing marker for equids (Han et al., 2010) and an HMS3 (~150 bp) microsatellite marker for horses (Guerin et al., 1994; Nielsen et al., 2007), using the extracted DNA obtained from both the protocols. We carried out PCR reactions using 10 μL of 2× Taq Mix Red PCR MasterMix (PCR Biosystems), 500 nM of each primer, 1–5 μL of DNA extracts for microsatellite/sexing and PCR grade water to reach a total volume of 20 μl. PCR conditions for ZFX‐ZFY included an initial denaturation (95°C for 2 min), 35 cycles of denaturation (95°C for 45 s), annealing (58°C for 40 s) and extension (72°C for 1 min). PCR conditions for HMS3 included an initial denaturation (95°C for 5 min), 40 cycles of denaturation (95°C for 30 s), annealing (60°C for 30 s) and extension (72°C for 30 s), followed by a final extension (72°C for 10 min). We ran a positive control of the DNA extracted from blood and a negative control in each PCR run. We amplified the sex and microsatellite markers three times independently for each sample. The evaluation of success rate is provided below.

#### 2.4.2 Assessment of wild‐ass DNA quantity through qPCR amplification of GAPDH from the two protocols

We quantified the Asiatic wild‐ass DNA by amplifying the housekeeping gene GAPDH from both extraction types (Kaczensky et al., 2011), using the SYBR Green Detection System in a CFX96 Touch Real‐Time PCR Detection System. We performed each qPCR in a 20‐μl reaction volume containing 5 μL of DNA, 200 nM of each primer and 2× qPCRBIO SyGreen Blue Mix Hi‐ROX (PCR Biosystems) with the following cycle conditions: initial denaturation step at 95°C for 3 min; 40 cycles at 95°C for 5 s; annealing at 62°C for 20 s; with a final continuous fluorescence acquisition (65°C to 95°C in 0.5°C increments) for the melting curve analysis. We conducted an absolute quantification with external calibration using six serial dilutions of 1:5 (17.5, 3.5, 0.7, 0.14, 0.028, and 0.0056 ng) of a tissue DNA standard, previously quantified by absorbance (*A*_260_) in a qubit instrument (Invitrogen). We amplified samples and standards in triplicates and recorded the Mean *C*_q_ values for each sample for further comparisons.

### 2.5 Assessment of multiple parameters for the two protocol outputs at the level of diet DNA

#### 2.5.1 Amplification success of trnL marker through PCR amplification

We amplified part of the trnL intron in the plant chloroplast using c‐h primers (Taberlet et al., 2006). PCR reactions were carried out in 25 μL of reaction volume using 12.5 μL of 2× Taq Mix Red PCR MasterMix (PCR Biosystems), 9.5 μL of RNAase‐free water, 500 nM of primer and 1 μL of DNA extracts with conditions including an initial denaturation (95°C for 3 min), 35 cycles of denaturation (95°C for 30 s), annealing (55°C for 30 s) and extension (72°C for 30 s), followed by a final extension (72°C for 10 min). The amplifications were repeated three times independently, and negative controls were run in each experiment. The evaluation of the amplification success rate is detailed below.

#### 2.5.2 Amplicon library preparation and sequencing for metabarcoding of diet DNA

Library construction and sequencing were performed at the Jonah Ventures Laboratory in Boulder, Colorado, USA, for DNA metabarcoding with the c–h primers of the trnL intron in the plant chloroplast (Taberlet et al., 2006). After PCR amplification (conditions mentioned above), amplicons were cleaned with Exo1/SAP for 30 min at 37°C, followed by inactivation at 95°C for 5 min, and stored at −20°C. A second round of PCR was performed for inserting a unique 12‐nucleotide index sequence in each sample. The indexing PCR included Promega Master mix, 0.5 μM of each primer and 2 μL of template DNA (cleaned amplicon from the first PCR reaction); it then involved an initial denaturation of 95°C for 3 min, followed by eight cycles of 95°C for 30 s, 55°C for 30 s and 72°C for 30 s. Next, 25 μL of PCR amplicon was purified and normalised using the Life Technologies Sequal Prep Normalization Kit (cat no. A10510‐01) according to the manufacturer's protocol. Samples were then pooled together by adding 5 μL of each normalised sample to the pool. Sample library pools were sent for sequencing on an Illumina MiSeq in the CU Boulder BioFrontiers Sequencing Center using the v2 500‐cycle kit (cat no. MS‐102‐2003). Necessary quality control measures were performed at the sequencing centre prior to sequencing.

#### 2.5.3 Bioinformatics analysis

Sequencing success and read quality were verified using FastQC v0.11.8 (Andrews, 2010), and reads were demultiplexed by using Illumina‐utils v2.6 (iu‐demultiplex) with default settings (BioProject PRJNA944116). Sequences of each sample were then merged using the ‐fastq_mergepairs option in Usearch v11.0.667 (Edgar, 2010). The forward primer and reverse primer were removed along with sequences that were less than 108 bp, using Cutadapt v1.18 (Martin, 2011). Expected error filtering, as implemented in Usearch, discarded low‐quality reads (max_ee = 0.5) (Edgar & Flyvbjerg, 2015). In addition, reads affected by sequencing and PCR errors were then removed using the unoise3 algorithm with an alpha value of 5 (Edgar, 2017). This denoising was applied to each individual sample, and the ESVs were compiled in an ESV table, including sequences and read counts for each sample. Taxonomy was assigned to each ESV by mapping them against a GenBank reference database (Benson et al., 2005), as well as Jonah Ventures voucher sequence records (Craine, 2021), using usearch_global with –maxaccepts 0 and –maxrejects 0 (Craine, 2021) to ensure mapping accuracy. Consensus taxonomy was generated by first considering 100% matches, and then going down in 1% steps until hits were present for each ESV.

#### 2.5.4 Assessment of diet parameters obtained from metabarcoding data

For the diet dataset of amplicon sequences, we retrieved all the sequences assigned at the genus level (the taxonomic resolution that is available at the molecular level for most plants in the area) and compared the two protocols using the following five parameters: the average number of total reads at the genus level, the average number of ESVs assigned to a genus, the average number of genera, diversity index (Shannon‐Wiener Index) and vegetation compositional difference at the genus level. All calculations were performed using Microsoft Excel and Vegan Package in R software (v4.2.2; R Core Team, 2021).

### 2.6 Statistical analysis and selection of a protocol

For PCR success rates of sex‐linked marker, microsatellite (target species) and trnL marker (diet), amplification success was scored manually for each amplification output based on gel band intensity and clarity: It was scored as 2 for strong amplification, 1 for weak amplification and 0 for no amplification. An average value for three repeats of each sample was considered as the final score for amplification success for the marker per sample, and then, it was averaged across samples for each protocol (Vo & Jedlicka, 2014). For quantitative comparisons of GAPDH amplification by qPCR, we evaluated the Cq value of each sample. We applied the Wilcoxon signed‐rank test in R software (v4.2.2; R Core Team, 2021) to infer significant differences in DNA concentration and amplification of target species and diet markers, as well as GAPDH quantification between the two protocols.

For diet data comparison between two protocols, we used the Wilcoxon signed‐rank test, implemented in R (v4.2.2; R Core Team, 2021) to assess the four different parameters: total reads at genus level, number of ESVs at genus level, number of genera obtained and richness index. We also conducted post hoc power analysis for our statistical tests for all the parameters (both focal species and diet) using the G*POWER software (paired Wilcoxon signed‐rank Test, α = 0.05) (Faul et al., 2007). Additionally, the relative read abundance/sample at the genus level comparison between the two protocols was presented in a heatmap, obtained using the PASTv4.3 software (Hammer et al., 2001).

We also conducted a normalised multi‐dimensional scaling (NMDS) multivariate analysis to represent the differences in the dietary findings at the finest‐scale resolution, that is the genus level. We performed the NMDS analysis using the Scatterplot3d and Vegan packages in R (Cavender et al., 2014; Clarke, 1993; Oksanen et al., 2020) and tested the significance in the difference in genus composition between the groups (protocols) using a bootstrapping PERMANOVA test (Anderson, 2017). To assess the sample adequacy (power) of PERMANOVA test, we evaluated the pseudo multivariate dissimilarity‐based standard error (MultSE) versus sample size based on Bray–Curtis dissimilarities using the multSE function (MSEgroup.d) in R software (10,000 resamples) (Anderson & Santana‐Garcon, 2015).

Finally, based on the statistical outputs for 11 parameters (4 for the target species, 5 for the diet and 2 for general), we chose the protocol which performed better for majority of the parameters.

## 3 RESULTS

### 3.1 Concentration and purity of DNA obtained from Protocols A and B

**FIGURE 3:**
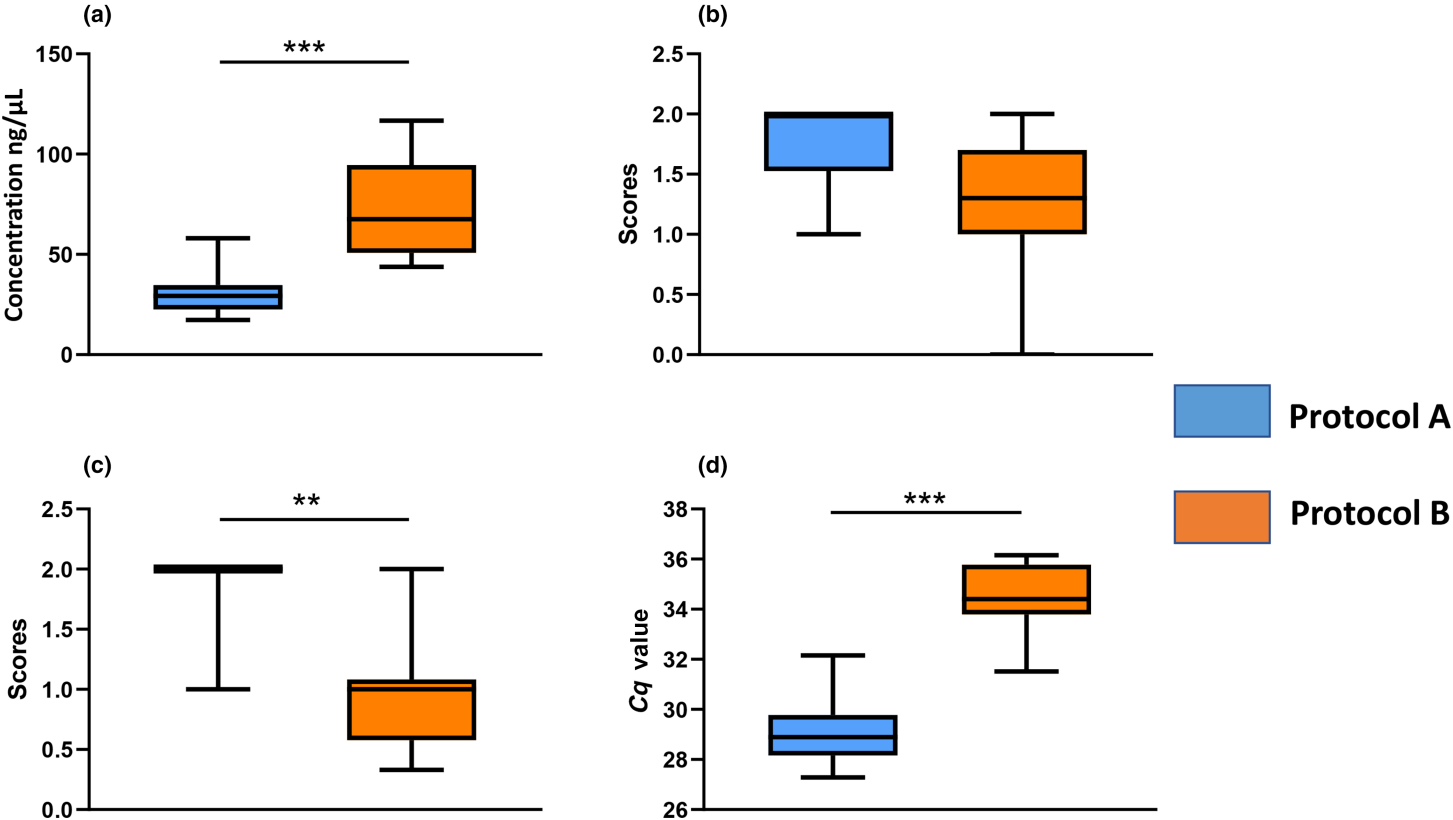
Comparison between performances of Protocol A and Protocol B assessed via DNA concentration (Panel a), index of PCR success for sex marker across triplicate PCRs (Panel b), index of PCR success for microsatellite marker across triplicate PCRs (Panel c), and the *C*_q_ values obtained from a qPCR of the GAPDH gene (Panel d). The upper and lower boundaries of the box plot represent the maximum and minimum values, the box represents the interquartile range, and the horizontal line within the box represents the median value. The statistical significance, if present, is shown by *** or ** representing *p* ≤ .001 or *p* ≤ .01.

Assessment of the DNA concentration by spectrophotometer values revealed that the samples (*n* = 10) extracted by Protocol B had significantly higher average concentrations (72.43 ± 24.18 ng/μL, $\bar{X}$ ± SD) than Protocol A (32.62 ± 11.7 ng/μL, Wilcoxon signed‐rank Test, *W* = 0 and *p* = .001) (https://figshare.com/s/48e92c390b3ed856d355) (Figure 3a). The nanodrop 260/280 purity ratios of all samples, irrespective of extraction method, had ratios of ≥1.8, indicating very little contamination.

### 3.2 Comparative assessment of target species DNA obtained from Protocols A and B

In the case of sex‐linked and microsatellite markers, we found that Protocol A had better amplification success than Protocol B (sex‐linked marker average amplification score: 1.75 ± 0.36 vs. 1.31 ± 0.58; microsatellite: 1.8 ± 0.42 vs. 0.96 ± 0.48, respectively), but the differences were only significant in the case of the microsatellite marker (sex‐linked marker: Wilcoxon signed‐rank test, *W* = 5 and *p* > .05; microsatellite marker: Wilcoxon signed‐rank test, *W* = 1 and *p* < .05) (https://figshare.com/s/48e92c390b3ed856d355) (Figure 3b,c).

The quantity of the Asiatic wild‐ass DNA was significantly higher in Protocol A than in B, as verified by the *C*_q_ values of the housekeeping gene GAPDH in both extraction types (average *C*_q_ value 29.07 ± 1.38 vs. 34.52 ± 1.41, respectively, Wilcoxon signed‐rank test, *W* = 0, *p* = .001) (https://figshare.com/s/48e92c390b3ed856d355) (Figure 3d).

### 3.3 Comparative assessment of diet DNA from the two protocols

We found no significant difference in PCR‐based trnL amplification success rates between the two protocols, as all samples scored 2 irrespective of method.

**FIGURE 4:**
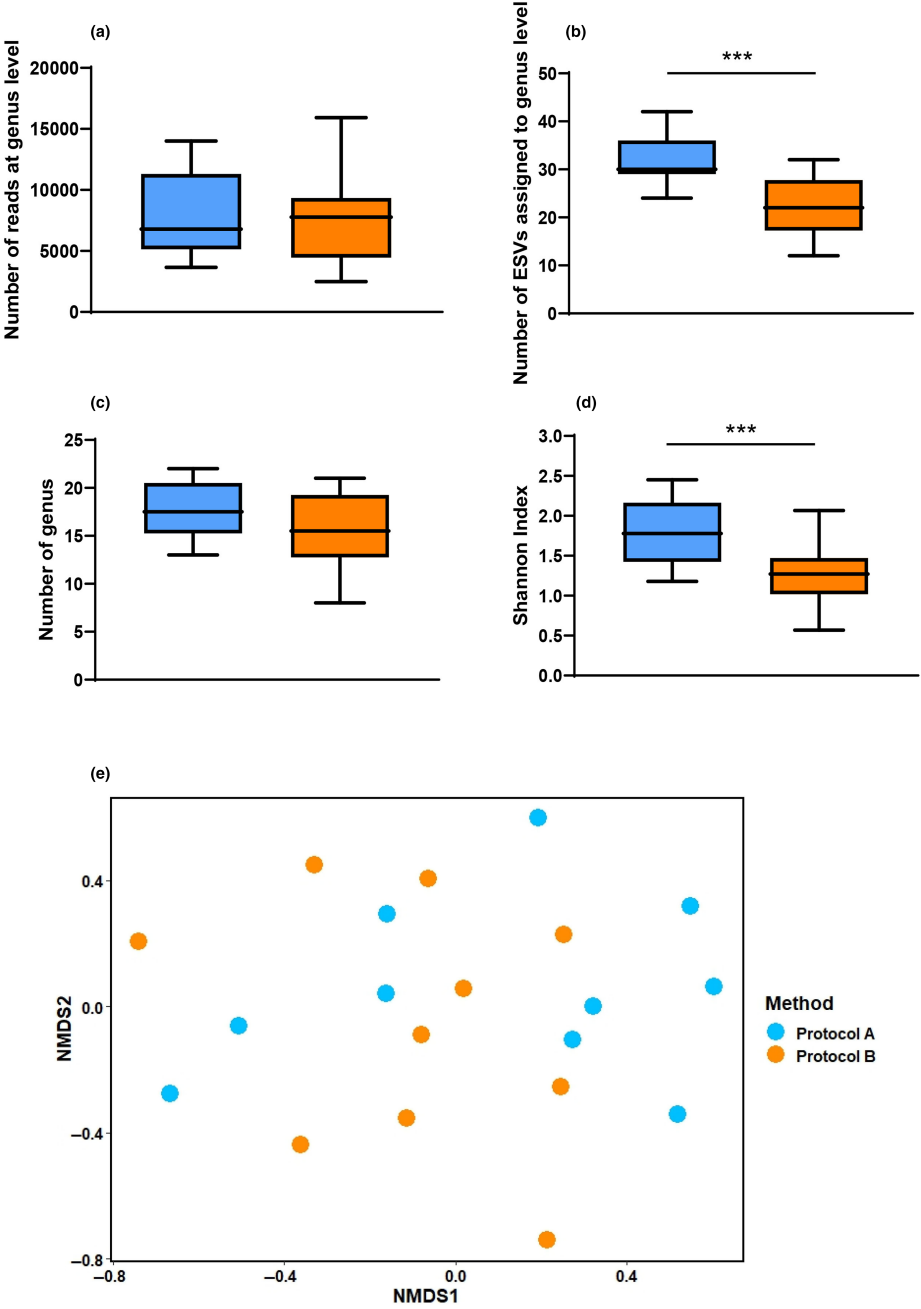
Assessment of Protocol A versus Protocol B at the genus level. Panel a: the total number of reads obtained at genus level from each protocol/sample; Panel b: the number of ESVs assigned to a particular genus; Panel c: the total number of plant genera recovered from each method; Panel d: Shannon Index of diversity at the genus level; and Panel e: two‐dimensional NMDS plot showing the genus composition comparison between the two protocols (*n* = 10 samples). The upper and lower boundaries of the box plot represent the maximum and minimum values, the box represents the interquartile range, and the horizontal line within the box represents the median value. The statistical significance, if present, is shown by *** or ** representing *p* ≤ .001 or *p* ≤ .01.

**FIGURE 5:**
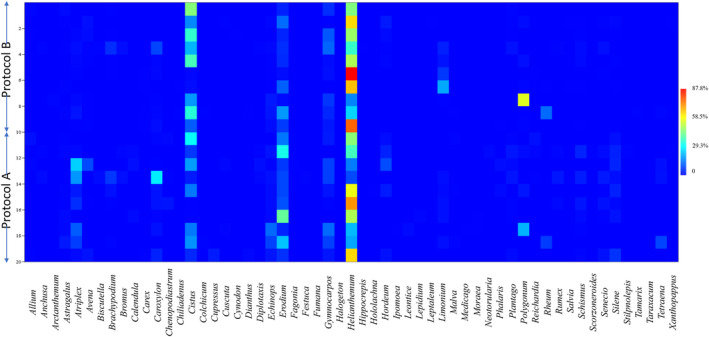
Heatmap of the relative read abundance (%) of each genus for each of the two protocols. The top 10 samples along the *Y*‐axis represent samples processed using Protocol B while the bottom 10 samples represent the same samples processed using Protocol A. The *X*‐axis represents the names of each of the identified genera. Colour gradient from blue to red indicates increasing relative read abundances.

The amplicon sequencing technique yielded an average of 14,264 ± 4867 and 13,126 ± 4272 reads from Protocols A and B, respectively, across all samples. We observed no significant differences in the average total number of reads obtained at the genus level between the two methods (Protocol A: 7451 ± 4058; Protocol B: 7869 ± 3653; Wilcoxon signed‐rank test, *W* = 27 and *p* > .05) (https://figshare.com/s/48e92c390b3ed856d355) (Figure 4a). The number of ESVs assigned to the genus level indicated that Protocol A had a significantly higher average value than Protocol B (31.8 ± 5.73 vs. 22.3 ± 6.44, respectively, Wilcoxon signed‐rank test: *W* = 2, *p* < .05) (https://figshare.com/s/48e92c390b3ed856d355) (Figure 4b). We retrieved an average of 17.6 ± 3.16 and 15.6 ± 4.08 genera from Protocol A and Protocol B, respectively (Wilcoxon signed‐rank test, *W* = 12 and *p* > .05) (https://figshare.com/s/48e92c390b3ed856d355) (Figure 4c). The relative abundances of all the genera in each sample obtained from both protocols are presented in a heatmap (Figure 5). Details of the relative read abundances of the genera (from both Protocol A and Protocol B) representing at least 1% of all reads in a single sample are given in Table 1. We obtained a total of 57 genera from both protocols, with Protocols A and B recording 49 and 44 genera, respectively. The genera obtained in Protocol A represented a total of 28 families while 24 families were represented in Protocol B, with 23 being common between them. *Helianthemum* was the dominant genus in both protocols, constituting an average of 50.8% and 38.42% of relative abundance reads for Protocols A and B, respectively. Additionally, we recorded an average higher value of the Shannon‐Weaver Index in Protocol A than in B (1.79 ± 0.43; vs. 1.27 ± 0.41, Wilcoxon signed‐rank test, *W* = 0 and *p* = .001) (https://figshare.com/s/48e92c390b3ed856d355) (Figure 4d). The post hoc power analysis for the parameters used to compare the two protocols yielded an average power of 0.78 (SD: 0.3) (Figure S2A). The multivariate analysis with NMDS ordination (stress value of 0.13, indicating a good representation of pairwise dissimilarity in reduced dimensions) showed no separation of samples between the two protocols based on plant genera composition (PERMANOVA value *p* > .01) (https://figshare.com/s/48e92c390b3ed856d355) (Figure 4e). In the case of sample adequacy (power) of PERMANOVA test, we found that the pseudo multivariate dissimilarity‐based standard error (MultSE) stabilised around sample size of 7–8 (Figure S2B).

**TABLE 1:** Relative read abundances (%) of genera representing at least 1% of all the reads generated at the genus level for a single sample (including both Protocols A and B). Reads representing less than 1% have been merged as ‘others’.

Sample	Method	Atriplex	Avena	Caroxylon	Cistus	Echinops	Erodium	Gymnocarpos	Helianthemum	Hordeum	Limonium	Polygonum	Rheum	Schismus	Silene	Tetraena	Others
7038AE	Protocol A	0.00	0.00	0.00	44.60	0.00	3.74	5.22	44.03	0.00	0.00	0.00	0.00	0.00	0.00	0.00	2.41
7037AE	Protocol A	0.00	1.51	0.11	15.75	0.65	12.34	0.29	62.77	2.75	0.00	1.24	0.00	0.16	0.69	0.00	1.73
7036 AE	Protocol A	0.67	1.19	0.00	34.84	0.00	4.03	9.99	46.95	1.12	0.00	0.00	0.00	0.27	0.20	0.00	0.74
7034AE	Protocol A	1.39	0.36	7.83	20.72	0.78	5.17	11.55	35.33	0.29	3.51	0.00	0.00	1.10	0.00	0.00	11.97
7032AE	Protocol A	0.78	0.27	0.58	37.18	0.27	4.75	1.15	49.14	0.63	2.00	1.51	0.00	0.98	0.29	0.00	0.46
7030AE	Protocol A	0.36	0.00	0.73	0.75	0.18	1.34	1.57	87.82	0.00	6.41	0.00	0.00	0.00	0.00	0.00	0.84
7029AE	Protocol A	0.00	0.00	0.08	1.31	0.16	10.87	0.00	65.85	0.00	19.28	0.65	0.14	0.18	0.12	0.00	1.37
7027AE	Protocol A	0.84	0.12	0.33	17.52	2.67	1.04	6.07	15.31	0.00	0.32	54.93	0.00	0.00	0.00	0.00	0.83
7024AE	Protocol A	2.17	0.18	0.68	33.51	2.19	17.53	4.30	24.55	0.00	0.00	0.14	12.18	0.00	0.00	0.59	2.00
7021AE	Protocol A	0.16	0.26	1.28	10.45	1.20	6.69	2.53	76.34	0.00	0.00	0.41	0.00	0.17	0.12	0.00	0.40
7038BE	Protocol B	0.00	0.00	0.61	30.01	0.00	13.53	2.94	45.03	1.15	0.00	0.00	0.00	0.00	1.54	0.00	5.19
7037BE	Protocol B	0.87	0.00	0.00	6.34	2.10	31.69	0.00	36.76	3.67	0.00	1.20	0.00	3.91	3.66	0.00	9.79
7036BE	Protocol B	23.94	8.35	0.32	18.05	0.47	6.35	7.22	14.38	8.49	0.00	0.00	0.00	0.00	3.98	1.49	6.96
7034BE	Protocol B	17.26	0.00	27.36	2.93	1.75	7.94	7.12	8.41	0.00	1.40	0.00	0.00	3.12	1.45	1.92	19.34
7032BE	Protocol B	2.64	0.00	2.04	13.83	1.25	7.97	2.10	56.51	2.88	0.93	2.40	0.00	0.00	3.55	0.00	3.90
7030BE	Protocol B	5.12	0.22	2.03	0.22	1.25	9.26	0.41	70.61	0.00	1.66	0.00	0.00	2.24	0.83	0.00	6.15
7029BE	Protocol B	0.00	0.00	0.45	0.24	0.98	40.80	0.00	49.13	0.00	1.71	0.00	0.00	0.94	2.05	0.00	3.70
7027BE	Protocol B	10.50	0.00	1.73	13.87	12.13	4.11	12.10	21.44	0.00	0.00	20.51	0.00	0.35	0.00	0.00	3.27
7024BE	Protocol B	6.91	0.00	0.00	16.82	7.40	22.42	7.27	19.01	0.00	1.49	0.00	6.84	0.00	0.00	7.85	3.98
7021BE	Protocol B	0.93	2.04	3.89	4.38	2.29	5.43	2.36	63.00	1.94	0.00	0.00	0.00	0.66	5.74	0.00	7.34

Overall statistical analysis of 11 parameters suggested that Protocol A performed better than B (details in Figure 1 and Figure S2) and was thus selected in our case study.

## 4 DISCUSSION

The increase in ‘combined studies’ that aim at exploring both the DNA of a target species and its diet calls for an efficient methodology relevant for both purposes. Here, we demonstrate a methodological framework for selecting a protocol that works well at the DNA level of both a large herbivore and its diet (Figure 1). Out of the two protocols tested, both yielded results for target species and diet DNA, but Protocol A performed better than Protocol B for most of the tested parameters.

The inherent variations in the success rate of non‐invasive genetic methods, owing to environmental and field conditions (Hájková et al., 2004; Murphy et al., 2000, 2002; Nsubuga et al., 2004), warrant a pilot study for ‘combined studies’, specifically testing the efficiency of different faecal sample processing methods (collection, preservation, extraction) in a specific research system (Beja‐Pereira et al., 2009; Goossens & Bruford, 2009). The most suitable protocol, based on the pilot study, can then be used for conducting large‐scale projects.

The first stage in the workflow of a pilot study for ‘combined studies’ is to select a few relevant protocols for the system of interest. The selection of the protocols should be based on environmental conditions in the field, the relative ease of execution, cost and wide applicability. In this study, we used two widely applied protocols for processing faecal samples for non‐invasive studies on herbivore species and their diet. Protocol A from this study has been extensively used for generating data from target species (e.g. Angom et al., 2020; Modi et al., 2021; Ramón‐Laca et al., 2015), and this protocol's extraction method has also been separately used in studies on species diet (e.g. Bhattacharyya et al., 2019; Galan et al., 2018; Guo et al., 2018; Shin et al., 2020). Protocol B (Qiagen Mobio Powersoil Kit, part of Protocol B) has been extensively used for recovering environmental DNA for dietary analysis (e.g. Ando et al., 2020; Craine, 2021; Jesmer et al., 2020; Pansu et al., 2019). As these two protocols have been previously used, mainly for their specific respective subjects (target species or diet), we wanted to test the efficacy of each of these protocols for generating both datasets simultaneously.

The next major challenge in a pilot study of ‘combined studies’ is to assess appropriate parameters that will enable making comparisons between the DNA outputs of the tested protocols at the level of both a target species and its diet. Firstly, we selected the same faeces for both protocols to nullify individual‐level variation for the estimated parameters. A basic step in comparatively assessing the two protocols is estimating the DNA concentration and purity (Hart et al., 2015; Vo & Jedlicka, 2014; Wen et al., 2016). However, assessing additional parameters is crucial, as it is reported that DNA purity does not ensure better downstream amplification and sequencing success (Hart et al., 2015). Next, to assess the protocols' efficacy for target species data, they should be tested by amplification of different types of target species markers, such as mtDNA markers, nuclear markers, and sex‐linked markers. Each type of marker can provide complementary information on the sample of the target species, such as on its sex, maternal line and heterozygosity. However, inherent differences in properties between the types of markers, the amount of DNA in the sample, primer sequence length, and copy numbers in the genome can influence the sensitivity of the protocols tested (Renan et al., 2012). In this study, we used, for the target species, two types of polymorphic markers: a sex‐chromosome‐linked marker and a microsatellite marker. Mitochondrial markers are comparatively easier to amplify than nuclear markers due to more copy numbers in the genome, and thus, we selected a nuclear marker for the comparison purposes (Ball et al., 2007; Kovach et al., 2003; Miller et al., 2003). Additionally, we also used a housekeeping mammalian gene (GAPDH) for quantifying the DNA concentration of the target species in both protocols. Such qualitative and quantitative multiple marker assessments provided a stronger, more robust inference about the tested protocols (Campbell & Narum, 2009).

Similarly, for the diet analysis, we selected a widely used and robust chloroplast marker trnL (Craine, 2021; Kartzinel et al., 2015; Mallott et al., 2018). It has been shown that trnL outperforms other similar markers, such as rbcl2, by generating more sequences and higher taxonomic resolutions in identifying plant families (Mallott et al., 2018). Apart from assessments of the amplification success of plant markers, a detailed quantitative bioinformatic examination of the sequences can provide fine‐scale comparisons between different protocol outputs. The comparison could be at any taxonomic level of interest, depending on the goals of the full research—for example plant families, genus, or ESVs—and depending on plant heterogeneity and genetic resolution. In this study, we conducted our comparative analyses using various parameters: total reads, reads assigned at the genus level, the number of ESVs assigned at genus level, the number and relative abundance of plant genera, diversity indices and the difference in genus‐level composition between the protocols. These parameters are widely used for providing qualitative and quantitative information about metagenomic DNA (Ando et al., 2020; Hart et al., 2015; Vo & Jedlicka, 2014). Since the same faecal sample has been subjected to two different protocols, a pairwise comparison of different parameters, using statistical approaches, was adopted (Hart et al., 2015; Vo & Jedlicka, 2014; Wen et al., 2016). The selection of the best protocol for the system of interest is thus dependent on a final comparative analysis that integrates both the DNA quality and quantity for both the target species and its diet.

Both protocols in our study yielded DNA of desirable purity (A260/280 ratio > 1.8). Similar observations have been reported in previous studies (Vo & Jedlicka, 2014). Nevertheless, Protocol A performed better for sex and microsatellite markers although the difference was statistically significant only for the microsatellite marker. Furthermore, quantitative evaluation of the target species DNA, through qPCR, revealed higher concentrations in samples extracted using Protocol A. The relatively better performance of Protocol A, with respect to the above‐mentioned parameters, may be explained by the presence of a large amount of sloughed epithelial cells of the target species on the faeces surface (Renan et al., 2012; Rutledge et al., 2009). However, the target species DNA was still successfully amplified in Protocol B, albeit at a lower efficiency than Protocol A, likely due to the presence of a higher inhibitor concentration and lower target species DNA inside of the faeces than on the outer surface (Ball et al., 2007; Waits & Paetkau, 2005).

For diet assessment, we found similar strong PCR amplifications of trnL from both protocols. This is probably because diet DNA is present across the entire faeces (both on the surface and inside) (Ando et al., 2020; Waits & Paetkau, 2005). Our metagenomic analysis of diet contents, obtained from both protocols, revealed that Protocol A generated a greater number of genera than Protocol B. In earlier studies, stool kit methods were found to generate a larger number of sequences/OTUs (Evans et al., 2018). All the genera obtained from both protocols have been verified to be present in the study area. We also recorded the same dominant genus in both protocols, strengthening the efficacy of each in diet analysis. We recorded a higher Shannon Index for the data generated from Protocol A, but we did not find statistically different genus compositions between the two protocols in a multivariate test. Although the subtle differences in diet contents between the two protocols may be due to the possible effects of different methodologies, these differences can also happen due to inherent problems associated with inhibitor concentrations and pipet malfunction during equimolar sample pooling in library preparation (Vo & Jedlicka, 2014). However, to reduce potential biases arising from contamination issues, we were very careful in collecting samples and conducting pre‐ and post‐PCR operations in separate physical spaces.

One of the important factors in a pilot study is the validation of the analytical framework to justify adequacy of sample size. A pilot study generally involves an assessment and selection of relevant methods/protocols which can be used later for a large‐scale study. In our case, the pilot study aimed to provide information on the efficiency of the methods and not on biological aspects of the population. For such purposes, the range of sample size commonly used in non‐invasive genetic pilot studies is between 05 and 20 samples (Costa et al., 2017; de Flamingh et al., 2023; Hart et al., 2015; Jones et al., 2021; Vo & Jedlicka, 2014; Wang et al., 2019). In this type of non‐invasive genetic pilot studies, there are often limitations with respect to the number of samples that can be used, such as the availability and quality of samples and budget constraints. Hence, it is important to find the proper ‘balance’ between a feasible sample size for collection and the statistical robustness in the information it provides. We suggest that the balance can be achieved by a combination of faecal sample design, comparison of different protocols in a paired design and assessment of multiple parameters to compare between the different protocols (as demonstrated in our case study). Moreover, in our suggested protocol we aimed at keeping the overall costs relatively low (e.g. ~65$/sample in our case for sample collection, storage, processing and sequencing) to ensure that these preliminary surveys, which we consider as crucial for an efficient study, will not be neglected. In our case study, we aimed to collect and use only very fresh faecal samples as they are known to contain better‐quality DNA (Biswas et al., 2022; Costa et al., 2017; Renan et al., 2012). Additionally, we wanted to collect samples during the spring season, the flowering season in the Negev desert, to maximise the capture of different plants in the diet. However, the elusive nature of wild ass specifically in spring (as their roaming distances are larger during this season, Giotto et al., 2015) posed a challenge in fresh sample collection. To overcome these limitations, we designed our study to increase the statistical robustness of the outputs. Firstly, we tested the significance of all parameters in a paired sample design, that is, the same samples were used for Protocols A and B (reducing variability between samples) which is recommended for studies with relatively small sample size (Fradette et al., 2003). Secondly, we have assessed the comparisons of the two protocols based on 11 parameters (two common parameters, three parameters of target species and six parameters of diet) to determine the best protocol suited to our system. (Viltrop et al., 2010). However, even with 10 samples, we obtained an average power of 0.78 ± 0.32 (for the analysed parameters), which is considered high enough (Brydges, 2019; Cohen, 1988). Additionally, the pseudo multivariate dissimilarity‐based standard error (MultSE) estimates for PERMANOVA tests (used for assessing difference in plant genus composition in diet) also supported sample adequacy in our case study. As we recommend other studies to follow the suggested framework, an initial pilot study with similar experimental design should pave a way to select a protocol based on statistical robustness that can be used to generate data at the population level. Although the most efficient protocol found in our pilot study for the wild ass in the Negev Desert was Protocol A, Protocol B also produced significant outcomes for both target species and diet, demonstrating that either approach may be utilised in studies to reliably describe the diet of herbivores. Even though we used faecal material from inside the faecal bolus for Protocol B, it surprisingly produced data for target species specifically. This may be due to fewer PCR inhibitory compounds in the diet or the aridity of the study site where DNA degrades more slowly than in moist environments (Kovach et al., 2003). The greater concentration of the DNA extracted by Protocol B is advantageous, especially when additional troubleshooting steps can rapidly deplete extraction stocks and consequently reduce their applicability for multiple usage. As different circumstances might give the advantage to a specific protocol over another, a pilot study involving multiple protocols is recommended, which will aid in selecting a favourable protocol that will work for ‘combined studies’ in herbivores. Furthermore, optimisation of standard protocols by specific adjustments for ‘combined studies’ could be tested in a designed experimental setup via additional pilot studies (Renan et al., 2012).

One of the most important aspects of a single unified protocol is its time and cost effectiveness (Hart et al., 2015). In ‘combined studies’, separation of protocols for target species and diet incurs extra time and cost. The average current cost and time associated with the extraction of 10 samples by Protocol A is 113 EUR and 2 h, while it is 102 EUR and 2.5 h for Protocol B. Thus, using a single optimised protocol for answering both types of questions in ‘combined studies’ may reduce the time and cost by half, rather than using two different protocols for this purpose. This may increase the feasibility and implementation of ‘combined studies’ and enable researchers to ask various questions simultaneously related to the genetics, genomics and diet of a species using non‐invasive samples. The same strategy for selecting one unified protocol could be useful for other sorts of ‘combined studies’, such as studies on target species and their gut microbiome, resistome and viral and parasitic load (Cao et al., 2020; de Flamingh et al., 2023; Franz et al., 2022; Srivathsan et al., 2016). With the increasing application of NGS to non‐invasive genetic projects, it is of paramount importance that standardised methods of sample processing for answering different questions be continuously updated. We hope that the strategies and workflows proposed in this study will highlight the importance of cost and time‐effective non‐invasive sample processing methods when designing ‘combined studies’.

## AUTHOR CONTRIBUTIONS

**Shrutarshi Paul:** Data curation (lead); formal analysis (lead); investigation (equal); methodology (equal); software (lead); visualization (lead); writing – original draft (lead); writing – review and editing (equal). **Naama Shahar:** Data curation (equal); formal analysis (supporting); investigation (equal); methodology (equal); software (supporting); validation (equal); writing – original draft (supporting); writing – review and editing (supporting). **Merav Seifan:** Conceptualization (lead); data curation (supporting); formal analysis (supporting); funding acquisition (lead); investigation (equal); methodology (supporting); project administration (lead); resources (lead); software (supporting); supervision (lead); validation (equal); visualization (supporting); writing – original draft (supporting); writing – review and editing (equal). **Shirli Bar‐David:** Conceptualization (lead); data curation (supporting); formal analysis (supporting); funding acquisition (lead); investigation (equal); methodology (supporting); project administration (lead); resources (lead); software (supporting); supervision (lead); validation (equal); visualization (supporting); writing – original draft (supporting); writing – review and editing (equal).

## CONFLICT OF INTEREST STATEMENT

The authors declare no conflict of interest.

### OPEN RESEARCH BADGES

This article has earned an Open Data badge for making publicly available the digitally‐shareable data necessary to reproduce the reported results. The data is available at excel files for the parameters analysed and codes for NMDS in ‘Figshare’ repository (https://figshare.com/s/48e92c390b3ed856d355). The demultiplexed raw unfiltered reads have been archived in GenBank (BioProject PRJNA944116, https://www.ncbi.nlm.nih.gov/bioproject/?term=PRJNA944116).

## BENEFIT‐SHARING STATEMENT

Benefits from this research accrue from the sharing of our data and results on public databases as described above.

## Supporting information


Figure S1

## Data Availability

We have archived the data (excel files and r codes used in our analysis) in ‘Figshare’ repository (private link‐https://figshare.com/s/48e92c390b3ed856d355). The demultiplexed raw unfiltered reads have been archived in GenBank (BioProject PRJNA944116).
