# Supplementary material for: An experimental design for obtaining DNA of a target species and its diet from a single non‐invasive genetic protocol
Source: Ecol Evol. 2023 Oct 22;13(10):e10616. doi: 10.1002/ece3.10616 (PMC10590962; doi:10.1002/ece3.10616)
Supplement: Supplementary file 1 — Figure S1 [file ECE3-13-e10616-s001.docx]

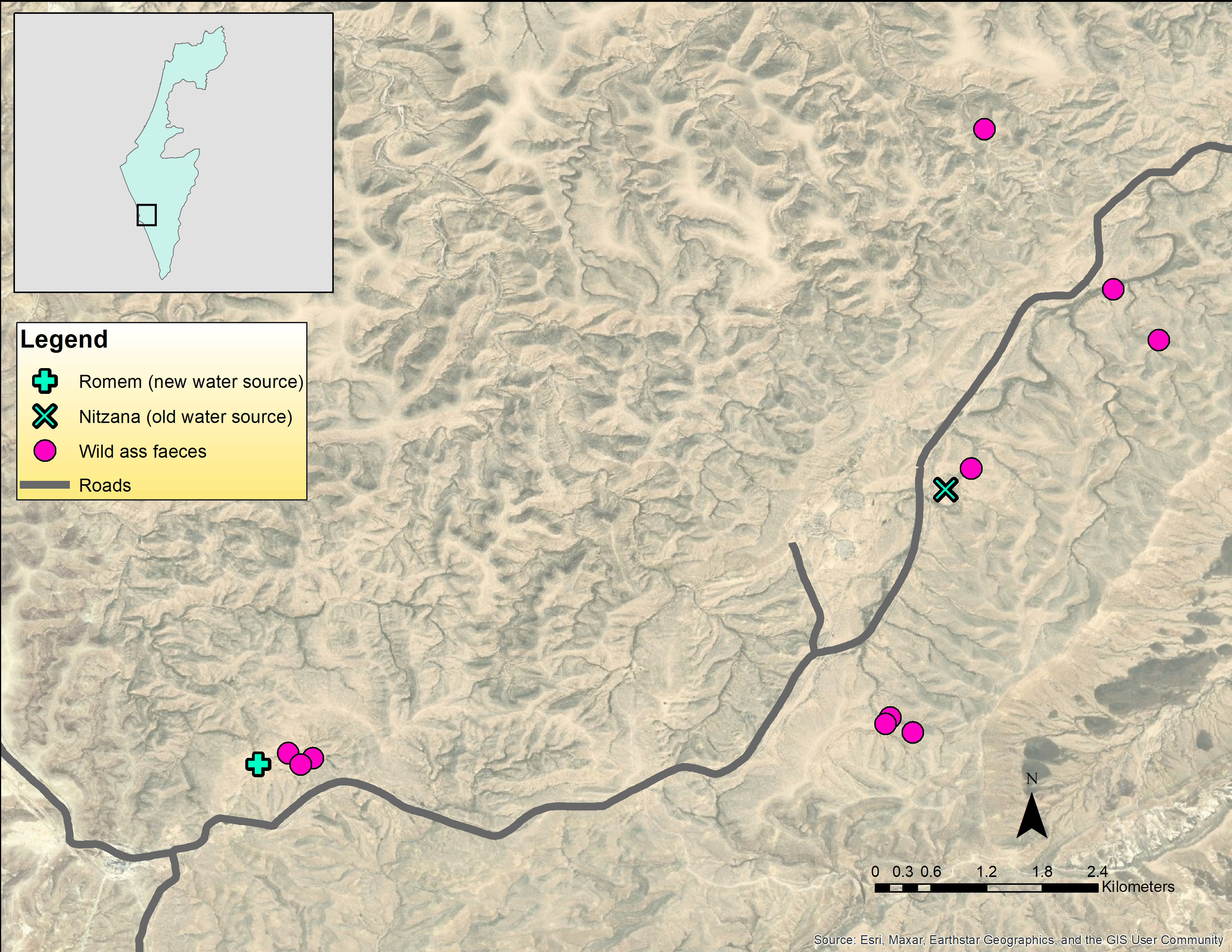


Figure S1: The fecal sampling locations (n=10) between the old water source in Nitzana and the new water source in Romem in the Negev Highlands, Israel


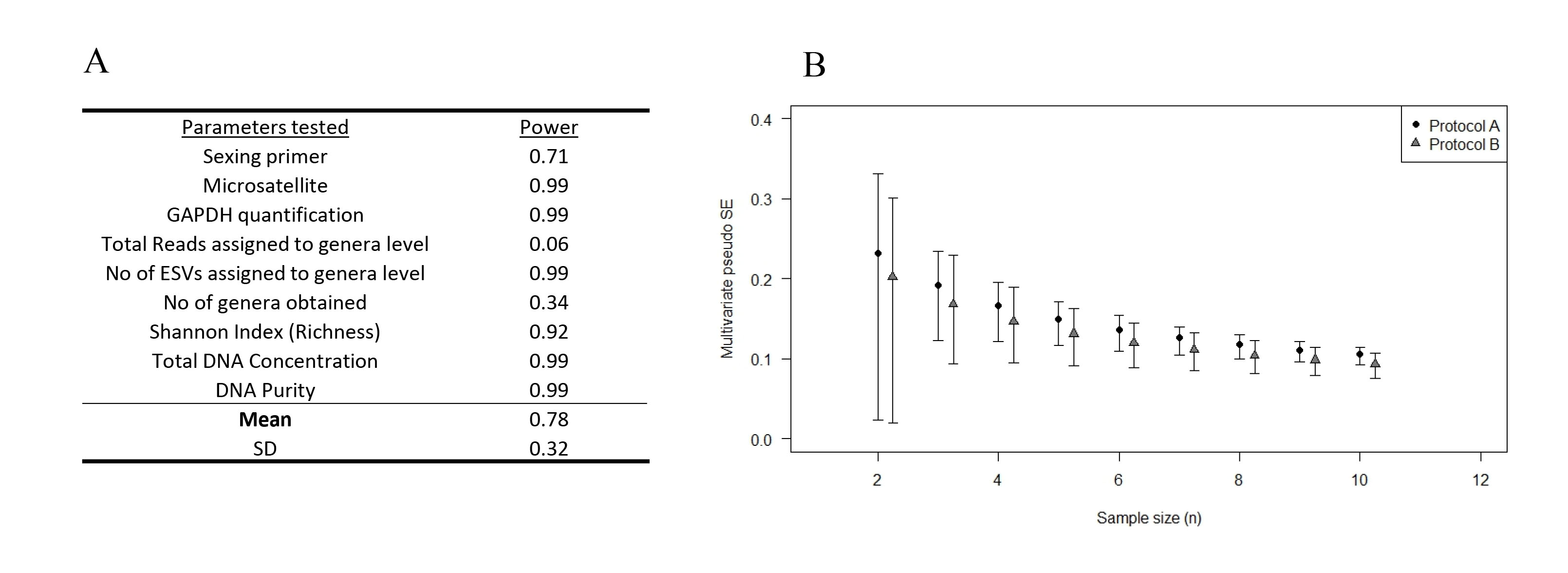


Figure S2: Outputs of posthoc power analysis (sample adequacy) conducted on all the parameters used to compare Protocols A and B. Panel A: Posthoc power obtained for each tested parameter (univariate) along with average and standard deviation Panel B: Multivariate pseudo standard error (MultSE) versus sample size for PERMANOVA test (multivariate) on the basis of Bray–Curtis dissimilarities calculated on abundance data using the double resampling method, with permutation-based means and bias-adjusted bootstrap-based error bars (10000 resamples for each).
